# Bruxism in awake dogs as a clinical sign of forebrain disease: 4 cases

**DOI:** 10.1111/jvim.16570

**Published:** 2022-11-02

**Authors:** Theofanis Liatis, Megan Madden, Katia Marioni‐Henry

**Affiliations:** ^1^ Queen Mother Hospital for Animals, Royal Veterinary College University of London Hatfield UK; ^2^ Hospital for Small Animals, Royal (Dick) School of Veterinary Studies University of Edinburgh Midlothian UK

**Keywords:** diencephalon, jaw clenching, mandible thrusting, teeth grinding, thalamus

## Abstract

**Background:**

Bruxism is a repetitive masticatory muscle activity characterized by clenching or grinding of the teeth, or by bracing or thrusting of the mandible, or both.

**Objectives:**

To investigate whether bruxism in awake dogs could be associated with brain lesions.

**Animals:**

Four dogs with episodic bruxism in the awake state.

**Methods:**

Observational retrospective single‐center case series. Inclusion criteria were dogs examined between 2010 and 2021 with episodic bruxism as a presenting complaint or observed during the examination or hospitalization, complete medical records and magnetic resonance imaging or computed tomography of the brain. Bruxism during epileptic seizures as oroalimentary automatism was an exclusion criterion.

**Results:**

Four dogs met the inclusion criteria. Two dogs had bruxism while awake as a presenting complaint, whereas in the remaining 2 it was a clinical finding. All dogs had neuroanatomical localization consistent with a forebrain lesion, with diencephalic involvement in 3/4. The diagnostic evaluation was consistent with neoplasia (n = 2) and meningoencephalitis of unknown origin (n = 2), in 1 case accompanied by corpus callosum abnormality affecting the forebrain, in 3 dogs advanced imaging findings were suggestive of increased intracranial pressure. All dogs were euthanized.

**Conclusions and Clinical Importance:**

Our results suggest that the presence of bruxism in the awake state associated with other neurological deficits might indicate a forebrain lesion.

**Abbreviations**
AB: awake bruxism
CCA: corpus callosum abnormality
CSF: cerebrospinal fluid
CT: computed tomography
FLAIR: fluid‐attenuated inversion recovery
MRI: magnetic resonance imaging
MUO: meningoencephalitis of unknown origin
NCL: neuronal ceroid lipofuscinosis
SB: sleep bruxism

## 1 INTRODUCTION

In humans, bruxism is a clinical sign representing repetitive masticatory muscle activity characterized by clenching or grinding of the teeth, bracing, or thrusting of the mandible or both.^1^ Bruxism has 2 distinct circadian manifestations: it can occur during sleep (indicated as “sleep bruxism” [SB]) or during wakefulness (indicated as “awake bruxism” [AB]). In humans, SB is very common and has been associated with socio‐psychological factors (e.g., anxiety, stress), whereas AB is considered rare.^2^ Bruxism in the awake state can be physiological when it occurs in an otherwise healthy individual,^3^ or pathological and further subdivided into primary (idiopathic; i.e., associated with disturbances in central neurotransmitter systems) or symptomatic (i.e., secondary to structural central nervous system lesions).^2^, ^4^ Bruxism in the awake state is a clinical sign that has been rarely investigated in veterinary medicine.

In humans, primary AB has been described without underlying disease but in the presence of a variety of predisposing factors such as anxiety, smoking, alcohol, genetics, or dental disease (occlusal interferences).^5^ Secondary AB has been reported in relation to medications or neurologic disease such as congenital encephalopathies (e.g., Down and Rett syndromes), cerebrovascular disease (e.g., anoxia, intracerebral hemorrhage, hypoxic or ischemic encephalopathy, acute ischemic stroke), encephalitis, traumatic brain injury, hydrocephalus, movement disorders (e.g., cranial and cervical dystonia, chorea), epilepsy, neurodegenerative disorders (e.g., Huntington's disease, multiple system atrophy, Alzheimer's disease) and brain tumor.^2^, ^4^, ^5^, ^6^, ^7^, ^8^, ^9^, ^10^ Clinically, acute onset of severe teeth grinding that occurs mainly during wakefulness and that cannot be prevented voluntarily should alert the clinician to the possibility of a secondary etiology of bruxism.^7^ Of interest, bruxism occurring secondary to neurological disorders is almost always AB.^2^

In veterinary medicine, there is inconsistency in the terminology regarding AB. Bruxism in the awake state has been reported occasionally as “teeth grinding” in case reports describing a variety of neurological signs such as epileptic seizures (oroalimentary automatism),^11^ carbofuran intoxication,^12^ idiopathic hypersialosis,^13^, ^14^ neuronal ceroid lipofuscinosis,^15^, ^16^, ^17^ and globoid cell leukodystrophy.^18^ Bruxism during wakefulness in otherwise healthy dogs has been anecdotally considered a stereotypy, drawing a dubious comparison with primary AB in humans, with the commonly described stereotypical behavior observed in horses,^19^ and the stress‐related bruxism that healthy sheep manifest during handling.^2^ In other species, AB is considered an indicator of pain^20^, ^21^, ^22^, ^23^ but reports of a proven association between AB and pain in dogs are lacking.

Recently, we observed a close temporal association between the onset of AB and other neurological signs in a dog diagnosed with a forebrain lesion and investigated whether secondary AB could be associated with structural brain disease in dogs. Our main aim was to investigate whether secondary AB had been recorded as a clinical finding in a wider population of dogs with structural brain disease and to speculate on specific brain areas that may be involved in the manifestation of AB.

## 2 MATERIALS AND METHODS

Ours was an observational retrospective, single‐center, case series study conducted by searching electronic records between 2010 and 2021. Search terms included: bruxism, teeth grinding, and jaw clenching. Inclusion criteria consisted of (a) complete medical records, (b) episodic AB as a presenting complaint or observed during physical or neurological examination or hospitalization, and (c) magnetic resonance imaging (MRI) or computed tomography (CT) of the brain. Exclusion criteria consisted of oroalimentary automatisms during epileptic seizures. As electroencephalography was not performed, the criteria of exclusion for oroalimentary automatisms such as teeth grinding, chewing movements or teeth chattering included (a) nondistractible episodes, (b) impaired consciousness, (c) concurrent autonomic signs, (d) evolution into a generalized tonic‐clonic seizure and (e) pre‐ or postictal signs. Diagnostic imaging was performed using a high‐field MRI scanner (Philips Intera 1.5‐T Pulsar System, Philips Medical Systems, Guildford, England) and a 64‐slice helical CT scanner (Somatom Definition AS Siemens, Erlangen, Germany).

## 3 RESULTS

### 3.1 Animals

Four dogs met the inclusion criteria: 1 each of Labrador, Boxer, and Great Dane, and 1 mixed breed dog. Three dogs were female, 2 of which were spayed, and the remaining was a neutered male dog (Supplementary material S1).

### 3.2 Presenting complaints

The main reasons for referral included loss of learned behavior (urinary and fecal accidents at home; n = 2), recent onset of episodic teeth grinding (n = 2), lethargy (n = 2), decreased or absent barking (n = 2), head turning (n = 1), body turning (n = 1), unilateral limb knuckling (n = 1), episodic head tremor (n = 1), and hypodipsia (n = 1; Supplementary material S1).

### 3.3 Clinical and neurological findings

Clinical examination was overall unremarkable apart from tachypnea (n = 3). Neurological examination identified episodic AB (n = 4), head turning (n = 3), head tilt (n = 2), pleurothotonus (n = 2), circling (n = 2), hyperesthesia upon head palpation (n = 2) and 1 each of: obtundation, low head carriage, thoracic limb hypermetria, hemiparesis, unilateral postural reaction deficits, hemineglect syndrome, menace response deficits, Horner syndrome, pupillary light reflex deficits, and episodic head tremor. Episodic AB was reported by the owners of 2/4 dogs as a sign of recent onset along with the other presenting complaints, whereas none of the owners reported episodic AB occurring historically in any of the dogs (4/4) before presentation. Neuroanatomical localization was consistent with a forebrain lesion in all 4 dogs, with diencephalic involvement suspected based on hemineglect syndrome, hypodipsia, hypernatremia and a combination of head turn and tilt without other vestibular signs.^24^, ^25^

### 3.4 Characteristics of episodic bruxism in the awake state

Bruxism during wakefulness was present in all dogs. In 2 dogs, AB had been noticed by the owners and was included in the presenting complaints, whereas in the other 2 dogs it was observed on admission by a veterinarian. In the first 2 dogs, AB was reported by the owners to occur mainly at rest, whereas in the other 2 dogs AB was observed during consultation. The main feature was grinding of the teeth accompanied by a characteristic squeaky or crunching sound. The AB was episodic and occurred randomly during the day, with no trigger having been identified. All dogs stopped the AB immediately if distracted. All dogs were conscious and responsive to their names during AB episodes. No autonomic or pre‐ and postictal signs were reported in association with AB in any of the dogs.

### 3.5 Clinicopathological findings

Clinicopathological findings were mainly nonspecific, apart from 1 dog (case 3) that had persistent hypernatremia which, in combination with a history of hypodipsia and abnormal neurological examination findings, was suggestive of a corpus callosum abnormality (CCA; Supplementary material S1).^26^

### 3.6 Advanced imaging findings

**FIGURE 1:**
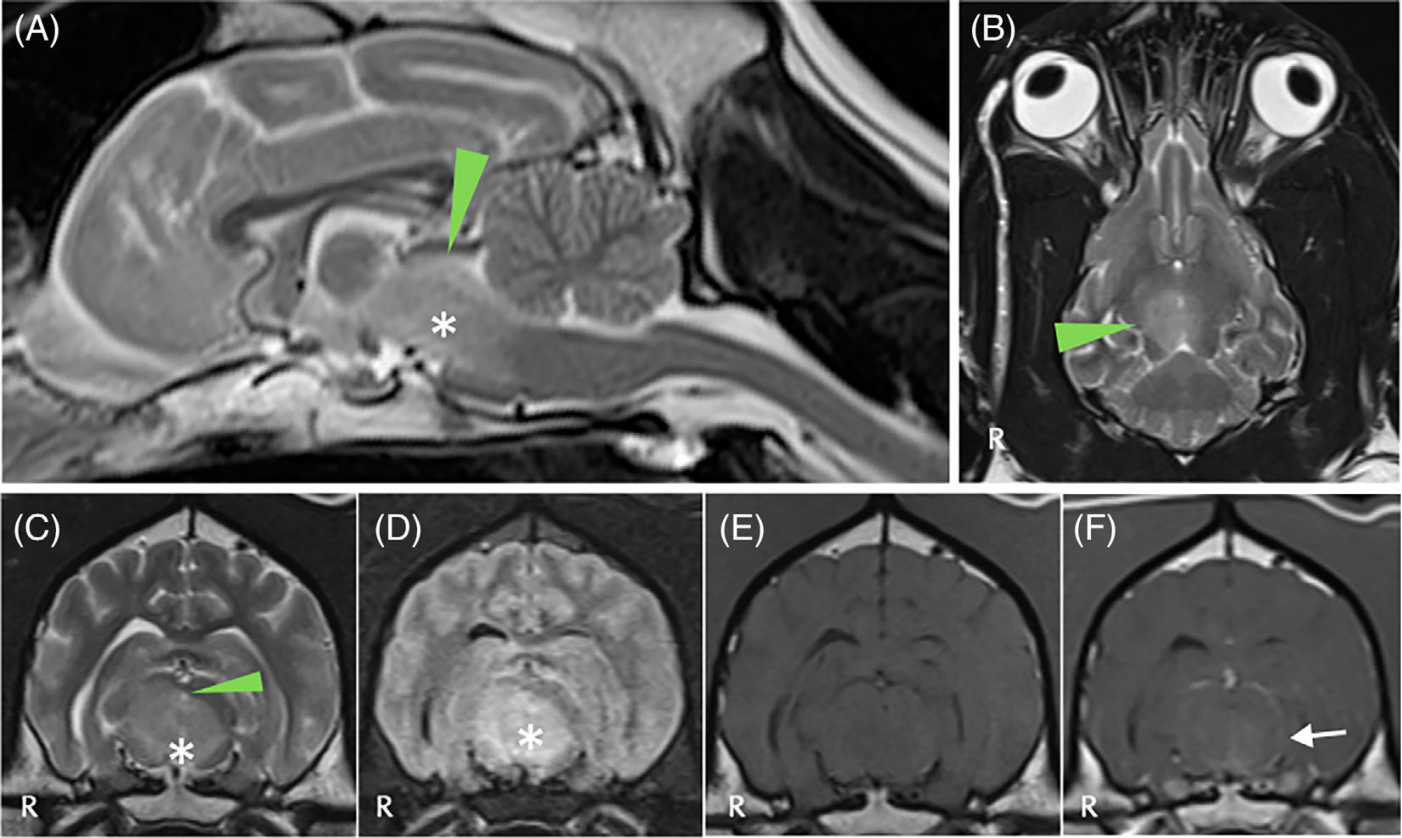
Magnetic resonance imaging of the head of dog 1 including T2W sagittal (A), T2W dorsal (B), T2W transverse (C), T2 FLAIR transverse (D), T1W precontrast (E) and T1W postcontrast (F) sequences, revealed a T2W and FLAIR hyperintense, T1W hypointense, poorly and heterogeneously contrast‐enhancing intra‐axial lesion (star) occupying a large area of the brainstem extending from the level of pons and occupying the majority of the midbrain (right‐side more severely affected) to the level of the thalamus/hypothalamus. Adjacent meningeal enhancement is observed (white arrow). Compression of the rostral colliculus and mesencephalic aqueduct is observed (arrowheads). The presumptive diagnosis was consistent with meningoencephalitis of unknown origin

**FIGURE 2:**
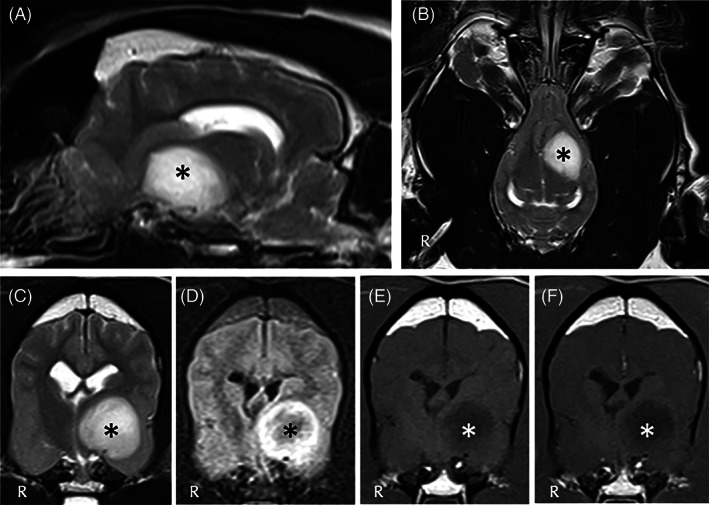
Magnetic resonance imaging of the head of dog 2 including T2W sagittal (A), T2W dorsal (B), T2W transverse (C), T2 FLAIR transverse (D), T1W precontrast (E), and T1W postcontrast (F) sequences, revealed large clearly defined rounded T2W hyperintense, T1W hypointense, FLAIR hyperintense with a slightly hypointense center, minimally contrast‐enhancing, intra‐axial mass lesion (star) centered over the left internal capsule and piriform lobe, extending into the frontal lobe and causing marked mass effect on the left thalamus/hypothalamus. There is also moderate midline and third ventricle shift to the right and left lateral ventricle compression. The diagnosis was suspected neoplasia (eg, glioma)

**FIGURE 3:**
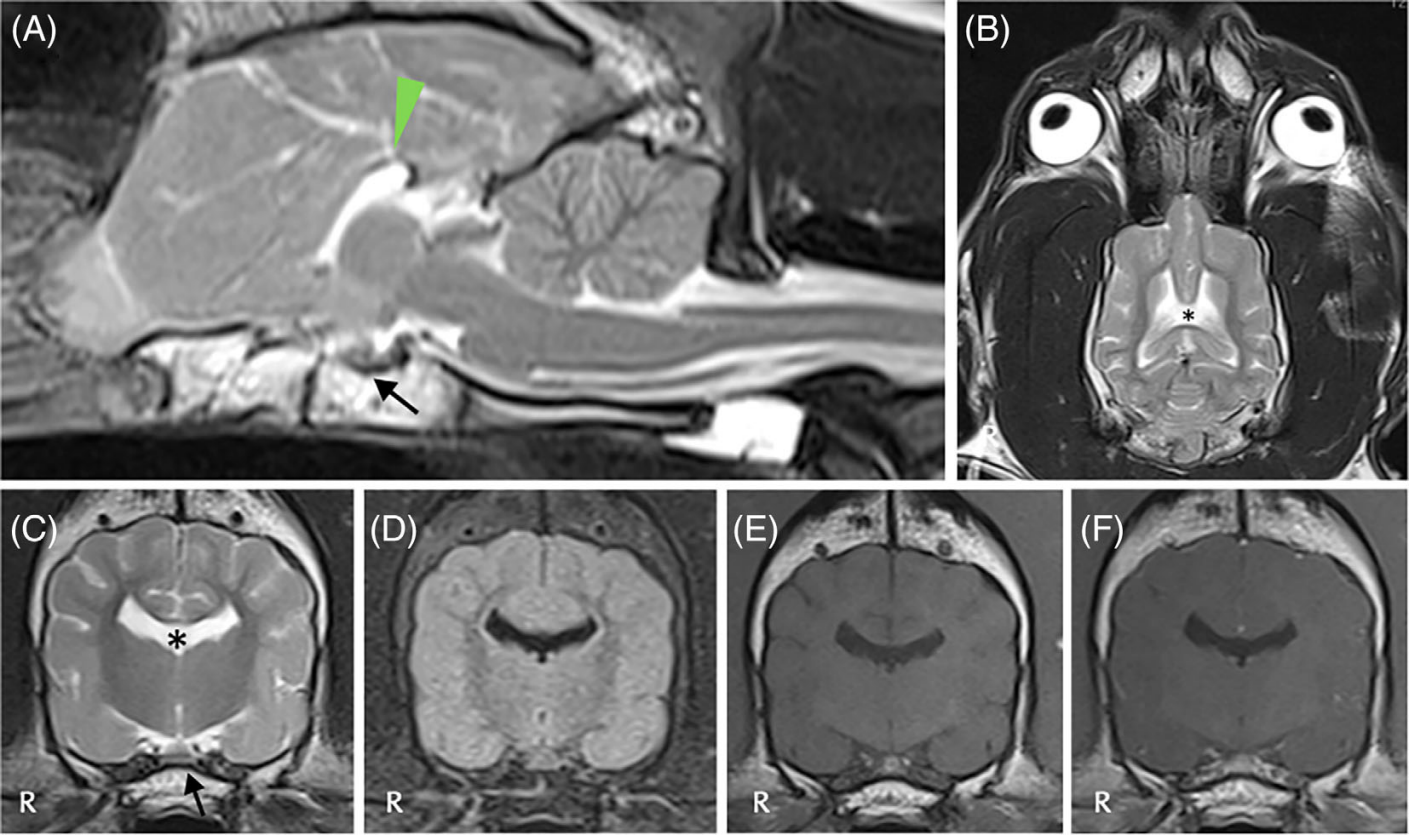
Magnetic resonance imaging of the head of dog 3 including T2W sagittal (A), T2W dorsal (B), T2W transverse (C), T2 FLAIR transverse (D), T1W precontrast (E) and T1W postcontrast (F) sequences, revealed absence of rostrum, genu and body of corpus callosum (arrowhead) with only a small portion of the splenium visible. The lateral ventricles have upturned pointed corners (upturned bat sign; star). The pituitary gland is subjectively small, with mildly increased CSF accumulation in the sella turcica (black arrow). Hypothalamus is T1W hypointense with a V‐shape. The diagnosis was congenital brain anomaly complex, whereas postmortem examination revealed also meningoencephalitis of unknown origin

**FIGURE 4:**
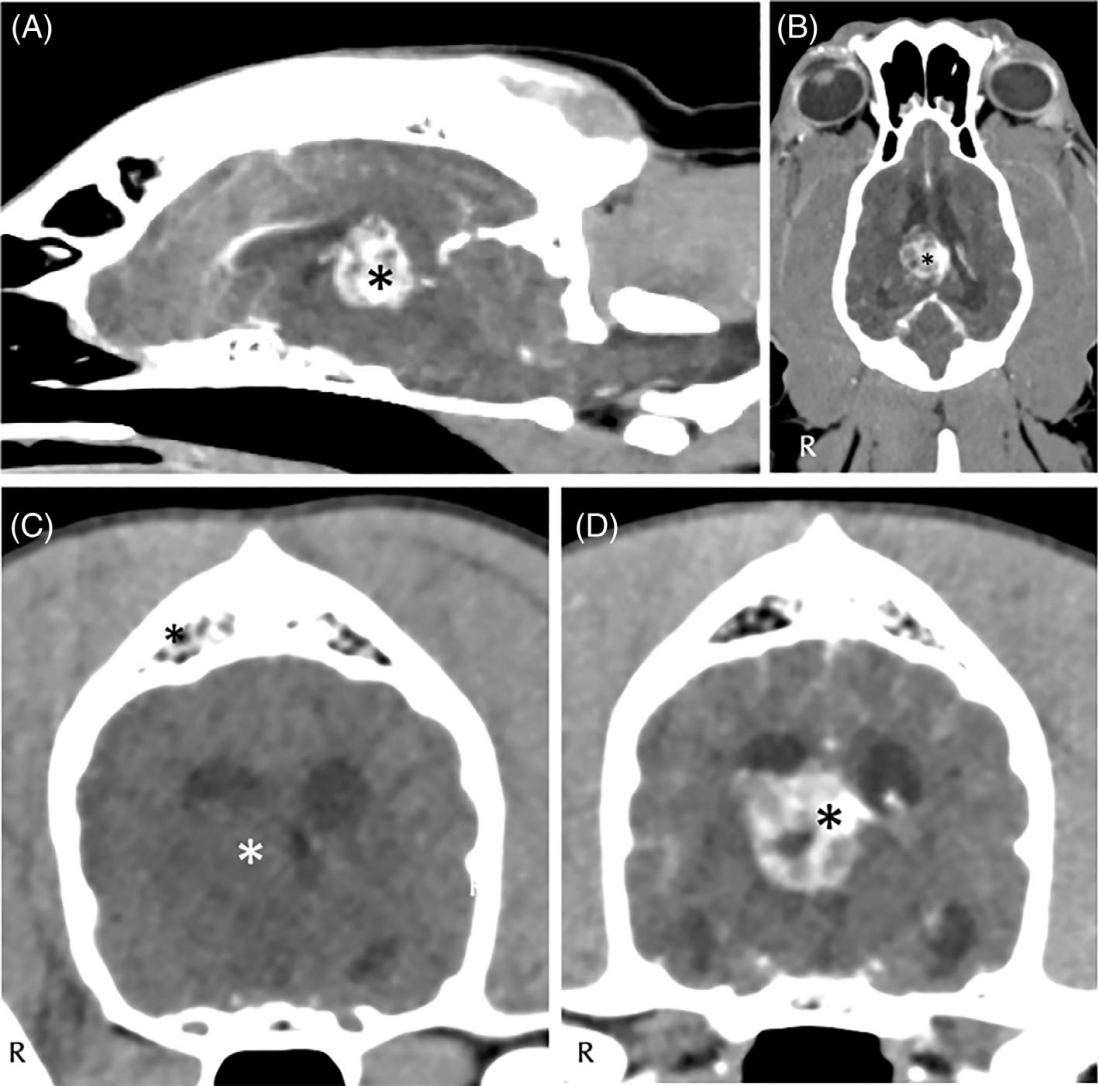
Computed tomography of the head of dog 4 at brain window including sagittal (A), dorsal (B) and transverse (C) precontrast sequences and transverse postcontrast images (D), identified a well‐circumscribed, irregularly marginated and strongly but heterogeneously contrast‐enhancing intraventricular mass lesion (star) located in the region of the third ventricle, closely associated with the medial aspect of the right lateral ventricle. This mass caused mild enlargement of the lateral ventricles and dilatation of the olfactory recesses. Asymmetry of the lateral ventricles with mild mass effect and distortion of the right lateral ventricle is evident. There is also mild dilatation of the mesencephalic aqueduct. There is a mass effect of the dorsal aspect of the right thalamus/hypothalamus and midbrain and a mild midline shift toward the left. A diagnosis of intraventricular mass (suspected neoplasia) was made

All dogs had lesions involving the forebrain (n = 4), with direct or indirect (ie, mass effect) involvement of the diencephalon in 3 and 1 cases, respectively. Advanced diagnostic imaging, including MRI (n = 3) and CT (n = 1), identified a diffuse ill‐defined intra‐axial lesion involving thalamus, hypothalamus, midbrain and pons (n = 1; Figure 1), a left‐sided well‐defined intra‐axial lesion that appeared to originate in the deep cerebral white matter and extending into the thalamus and hypothalamus, piriform and frontal lobes (n = 1; Figure 2), congenital brain anomaly complex including corpus callosum hypoplasia, septum pellucidum agenesis, partial empty sella, partial lobar holoprosencephaly, V‐shaped hypothalamus and occipital bone dysplasia (n = 1; Figure 3) and an intraventricular mass lesion closely associated with the third ventricle (n = 1; Figure 4). Signs of increased intracranial pressure were apparent on advanced imaging in 3/4 dogs, consisting of midline shift or displacement of the interthalamic adhesion (n = 2), lateral ventricle compression (n = 1), dilatation of olfactory recesses (n = 1), and caudal transtentorial cerebral (n = 1) and foramen magnum (n = 1) herniation.

More specifically, in case 1 (Figure 1) a T2W and fluid‐attenuated inversion recovery (FLAIR) hyperintense and T1W hypointense poorly and heterogeneously contrast‐enhancing intra‐axial lesion was observed involving the brainstem (pons, midbrain), thalamus and hypothalamus with interthalamic adhesion displacement. Differential diagnoses included meningoencephalitis of unknown origin (MUO) and less likely neoplasia (e.g., round cell neoplasia). In case 2 (Figure 2), a well‐defined rounded T2W hyperintense, T1W hypointense, FLAIR hyperintense with slightly hypointense center, minimally contrast‐enhancing intra‐axial mass was observed involving the left internal capsule, piriform lobe and frontal lobe with marked mass effect to the adjacent thalamus and hypothalamus. Differential diagnoses included neoplasia (e.g., glioma), and less likely MUO. In case 3 (Figure 3), a congenital brain anomaly complex was observed including corpus callosum hypoplasia, partial lobar holoprosencephaly, partial empty sella and occipital bone dysplasia with V‐shape of the hypothalamus. In case 4 (Figure 4), a well‐circumscribed irregularly‐marginated and strongly but heterogeneously contrast‐enhancing intraventricular mass lesion was observed in the region of third ventricle. All lesions had direct or indirect (i.e., mass effect) involvement of the diencephalon.

### 3.7 Cerebrospinal fluid analysis findings

Cerebrospinal fluid (CSF) analysis was performed in 1 dog and identified mixed pleocytosis with a total nucleated cell count of 3111 cells/μL (reference range, 0‐5 cells/μL) and total protein concentration of 233 mg/dL (reference range, <36 mg/dL). A presumptive diagnosis of MUO was made (case 1).

### 3.8 Clinical diagnoses

Clinical diagnoses included suspected MUO (case 1; Figure 1), intra‐axial brain mass (suspected glioma; case 2; Figure 2), congenital brain anomaly complex with primary adipsia (case 3; Figure 3), and intraventricular mass (suspected neoplasia; case 4; Figure 4).

### 3.9 Treatment and outcome

Treatment of suspected MUO (case 1) consisted of immunosuppressive doses of glucocorticoids (prednisolone) and cytosine arabinoside (cytarabine), whereas treatment of the suspected brain tumors (cases 2 and 4) included corticosteroids (n = 2) with the addition of lomustine in 1 case. Treatment of the dog with the congenital brain anomaly complex included desmopressin acetate and water supplementation via esophageal tube. All dogs were euthanized because of clinical deterioration (Supplementary material).

### 3.10 Necropsy examination

One dog (case 3) underwent necropsy that confirmed the suspected holoprosencephaly but also identified a chronic moderate and regionally extensive lymphohistiocytic meningoencephalitis affecting the rostral cerebrum, hippocampus and thalamus. Although infectious disease tests were not performed, given the signalment, complete vaccination status, lack of travel history and histopathological pattern, findings were consistent with granulomatous meningoencephalitis and therefore a clinical diagnosis of MUO.

## 4 DISCUSSION

In this case series, we report episodic AB as a clinical sign of forebrain disease in 4 dogs, 3 of which had more specific diencephalic pathology. We consider the pathophysiology of AB as a sign of central neurological disease, extrapolating from experimental studies and reports of human patients.

Pathophysiology and neural pathways of bruxism in humans and animals are not completely known. Early experimental studies in rats identified the medial hypothalamus as the area of the brain responsible for teeth chattering.^27^ Later, 2 hypotheses were developed for bruxism pathogenesis in humans: the peripheral cause and the central cause hypotheses. The peripheral cause hypothesis describes bruxism predominantly as a result of dental malocclusion.^28^ Other physiologic, anatomic, or psychosocial factors including gastroesophageal reflux, hypopnea, temporomandibular disorders, psychiatric disorders, alcohol and tobacco use, medications and high psychosocial stress also have been identified as risk factors for bruxism in the absence of central nervous system disease.^5^ The central cause hypothesis suggests that bruxism is a result of dysfunction of the cerebral cortex‐basal nuclei‐thalamus axis.^28^ Specifically, this axis controls motor preparation and execution of muscular movements in humans through direct and indirect pathways.^28^ Imbalance in the circuit processing of the basal nuclei might result in muscle hyperactivity and thus bruxism.^28^ Structural lesions of the thalamus and caudate nuclei (e.g., infarcts) could result in AB by disruption of both direct and indirect striatopallidal pathways leading to altered thalamocortical drive.^29^ In patients without structural disease, this imbalanced function of the basal nuclei has been hypothesized to be a result of aberrant development or function of the neural circuits underlying the cerebral cortex‐basal nuclei‐thalamic axis into a hyperkinetic movement disorder.^28^ In fact, a central bruxism generator complex has been proposed consisting of a neuronal circuit connecting striatum, subthalamus, thalamus and cerebral cortex.^2^ Disruption in this circuit creates a loss of inhibition of the higher cortical control on the trigeminal motor nuclei.^2^ The latter are located in the rostral pons and innervate the muscles of mastication which also are regulated by a complex network of inhibitory and excitatory inputs from the midbrain, pons, and medulla.^5^ The central role of the frontal lobe in bruxism is supported by several studies.^2^ Lesions of the frontal lobe may cause decreased cortical inhibition of the trigeminal motor nuclei located in the pons resulting in bruxism as reported in human patients with stroke, Hartnup disease, Rett syndrome, frontotemporal dementia, and normal pressure hydrocephalus,^2^, ^5^ but also with tumors.^9^ Also, AB might be a neurological marker of bilateral frontal cortical disease based on its high prevalence in diseases showing bilateral frontal dysfunction.^2^

In veterinary medicine, terminology describing teeth and jaw movements has been used inconsistently. The term bruxism has been used accurately to describe teeth grinding in case reports of dogs with neuronal ceroid lipofuscinosis (NCL),^15^, ^16^, ^17^ globoid cell leukodystrophy,^18^ corpus callosum abnormality with hypernatremia^30^ and carbofuran intoxication.^11^ Teeth grinding^13^ or jaw chattering^14^ have been reported in idiopathic hypersialosis. However, teeth grinding and chewing movements also are included in the description of oroalimentary automatisms in some types of epileptic seizures according to the International Veterinary Epilepsy Task Force consensus statement,^11^ similar to humans with temporal lobe seizures.^31^, ^32^, ^33^

In the dogs with AB and idiopathic hypersialosis, a limited diagnostic investigation was performed.^13^, ^14^ Neither of these dogs underwent advanced imaging and only 1 of the dogs had CSF analysis, which was within normal limits. Both dogs responded to phenobarbital treatment with cessation of AB and other neurological signs. Idiopathic (phenobarbitone‐responsive) hypersialosis has been proposed by some authors to represent an unusual form of limbic seizures.^13^, ^14^ Whether AB represents an oroalimentary automatism of limbic seizure, a movement disorder or oral pain in these cases remains unknown. However, in our cases, AB was not associated with hypersialosis or other features of temporal lobe epilepsy such as staring, mydriasis and facial twitching. Furthermore, dogs with oroalimentary automatisms associated with epileptic activity that could mimic bruxism were excluded.

Similar to humans, AB also has been reported as a clinical sign in dogs with central nervous system degenerative conditions such as neuronal ceroid lipofuscinosis (NCL),^15^, ^16^, ^17^ and in a miniature poodle with globoid cell leukodystrophy.^18^ Border collies with NCL appeared to manifest episodic AB but also episodic jaw chomping and limb myoclonus among other behavioral signs, motor abnormalities and loss of vision. A Terrier‐Poodle‐cross with NCL manifested progressive AB which was exaggerated by stress, along with vestibular signs, head tremor, behavioral signs, urinary and defecation accidents, night restlessness and barking.^16^ The dog underwent MRI of the brain which identified dilatation of the lateral, third and fourth ventricles, cerebral and cerebellar atrophy including decreased size of thalamus and basal nuclei and irregular shape and atrophy of the interthalamic adhesion. Neuronal ceroid lipofuscinosis was diagnosed on histopathology.^16^ The authors hypothesized that AB along with night restlessness and barking could be associated with the atrophied thalamus and basal nuclei detected on MRI.^16^ Bruxism in the awake state also has been reported in Dalmatians with NCL along with other behavioral signs such as aggression, cannibalism, and self‐mutilation.^17^ In the dogs in our cases series, the presence of a degenerative condition such as NCL or globoid cell leukodystrophy was considered unlikely, because 3 dogs were >4 years of age and had a relatively recent onset of clinical signs, and the remaining dog had necropsy findings that excluded the presence of NCL or globoid cell leukodystrophy.

No AB was observed in the largest study of CCA in dogs.^26^ Bruxism in the awake state has been described in a dog with CCA and in a horse with semilobar holoprosencephaly, both of which had concurrent hypernatremia, as reported in our case.^30^, ^34^ In humans, AB is a neurological sign of genetic diseases associated with CCA such as Rett syndrome.^35^, ^36^, ^37^, ^38^ In a recent case report of a dog with CCA, AB and other neurological signs were attributed to severe hypernatremia.^30^ The dog's MRI identified patchy FLAIR hyperintensities in the thalamus, a transient MRI finding previously reported in children with severe hypernatremia.^39^ In the dog reported here, given the necropsy findings of holoprosencephaly and concurrent meningoencephalitis, AB could be the result of the congenital anomaly complex, chronic meningoencephalitis affecting the forebrain including the thalamus or severe hypernatremia and transient thalamic changes, or a combination of factors.

Bruxism in the awake state also has been reported in several other species with a variety of central nervous system and metabolic diseases (Table 1). In goats, AB has been reported in thiamine deficiency and polioencephalomalacia,^40^ gliomatosis cerebri,^41^ cerebral coenurosis,^42^ cryptococcosis,^43^ and copper intoxication.^44^ In sheep, AB has been reported in NCL,^45^, ^46^ bovine spongiform encephalopathy,^47^ scrapie,^48^ bacterial meningoencephalitis,^49^ enterotoxemia,^50^ and tetanus.^51^, ^52^ Bruxism in the awake state also is considered a clinical sign of stress and pain in sheep, and therefore interpretation should be done carefully.^53^ In cattle, AB has been described in bovine herpesvirus encephalitis,^54^ bovine spongiform encephalopathy,^55^ and bacterial meningoencephalitis.^56^ In horses, AB is a clinical sign of Borna virus encephalitis,^57^ West Nile virus encephalitis,^58^ neural angiostrongylosis,^59^ and equine nigropallidal encephalomalacia.^60^ It is also a classic clinical sign of stomach ulcers in horses, and therefore gastrointestinal disease also should be considered in the differential diagnosis.^61^ Because of the retrospective nature of our study, we cannot completely eliminate the possibility of gastrointestinal disease in the dogs in our case series, but we are not aware of reports of AB as a manifestation of pain or gastrointestinal disease in dogs.

**TABLE 1:** Episodic awake bruxism and associated diseases based on previous veterinary literature including humans

Vitamin D	Dogs	Goats	Sheep	Bovine	Equine	Humans
Vascular						Cerebrovascular disease (ischemic and hemorrhagic)
Inflammatory/infectious	Meningoencephalitis of unknown origin	Cerebral coenurosis Cryptococcosis	Bovine spongiform encephalopathy Scrapie Bacterial meningoencephalitis Enterotoxemia Tetanus	Bovine herpesvirus encephalitis Bovine spongiform encephalopathy Bacterial meningoencephalitis	Borna virus encephalitis West Nile virus encephalitis Neural angiostrongylosis	Encephalitis
Traumatic/toxic	Carbofuran intoxication	Copper intoxication			Equine nigropallidal encephalomalacia	Traumatic brain injury
Anomalous	Corpus callosum abnormality and hypernatremia				Semilobar holoprosencephaly	Down and Rett syndrome Hydrocephalus
Metabolic		Thiamine deficiency and polioencephalomalacia				
Idiopathic	Idiopathic hypersialosis					Epilepsy Movement disorders
Neoplastic	Brain neoplasia	Gliomatosis cerebri				Brain neoplasia
Degenerative	Neuronal ceroid lipofuscinosis Globoid cell leukodystrophy		Neuronal ceroid lipofuscinosis			Neurodegenerative diseases (eg, Huntington's disease, multiple system atrophy, Alzheimer's disease)
Miscellaneous			Pain/stress		Gastrointestinal disease (gastric ulcers)	

The dogs described in our study manifested AB involving bilateral masticatory muscles despite the presence of lateralized lesions affecting the diencephalon (thalamus/hypothalamus) directly in 3 cases and indirectly (ie, mass effect) in 1 case. Interestingly, transcranial magnetic stimulation studies performed in humans during different biting tasks have determined that the corticotrigeminal projections to the masseter muscles are bilateral with a stronger contralateral projection providing a possible explanation for symmetrical clinical signs with a lateralized forebrain lesion.^62^ Additionally, in rats, inputs from the premotor area to the trigeminal nuclei and their connections within the pontine reticular formation are bilateral but with ipsilateral preponderance.^63^ Experimental studies in rabbits determined that unilateral stimulation of the limbic structures or the jaw area of the motor cortex could produce bruxism.^64^ In humans, sudden onset of AB associated with dysarthria was observed in a patient with an acute infarct in the right thalamus and chronic lacunar infarcts in the caudate nuclei on MRI and angiography.^29^ We hypothesize that in our cases the primary underlying cause led to dysfunction of the afferent or efferent or both pathways of the thalamus disrupting the normal interplay of neuronal circuits that connect the striatum, thalamus, subthalamus, and cerebral cortex and therefore creating a loss of inhibition of higher cortical control on the trigeminal motor nuclei located in the pons.^29^ Why AB is a rare clinical sign of forebrain disease in dogs remains unknown. It is possible that in veterinary medicine mild AB may be overshadowed by more severe neurological signs or behavioral abnormalities. Generally, AB is observed more frequently in large animals, which may be a result of awareness of large animal clinicians or handlers to AB as an indicator of pain in these species, or simply because of the size of the patient, which allows AB to be more audible. Similarly, in humans with thalamic or basal nuclei strokes, it is speculated that AB might be mild in most patients and hence awareness of this problem is low.^29^

Consistent terminology should be adopted in veterinary medicine regarding involuntary teeth and jaw movements. We propose the use of the term bruxism to describe the clinical sign of involuntary, vertical or lateral, clenching or grinding of the teeth by bracing or thrusting of the mandible, which is mainly audible as a characteristic squeaky or crunching sound and possibly visible with minor or no obvious movement of the jaw. Bruxism should not be confused with teeth chattering, which describes involuntary vertical movement of the jaw that can be a rhythmic mandibular tremor in corticosteroid‐responsive tremor syndrome^65^ or an episodic mandibular tremor in middle‐aged to old dogs that might be attributed to a trigemino‐trigeminal reflex^24^ and which we have observed in Cavalier King Charles Spaniels. Likewise, AB should not be confused with nonrhythmic movements (e.g., mandibular twitches as part of oroalimentary automatisms in epileptic seizures, jaw chomping as a sign of rapid eye movement behavioral sleep disorder or peripheral nerve hyperexcitability twitches in intoxications)^11^, ^24^, ^66^ or mandibular myoclonus, which refers to a shock‐like jerk of the mandible (e.g., neurological sequela of distemper).^24^ In AB cases (Video S1), the movement of the jaw usually is not so wide as to involve the majority of the masticatory muscles (i.e., obvious opening and closing of the jaw) which is why bruxism is clinically different from jaw chomping, teeth chattering and jaw tremor, twitches or myoclonus. The hallmark of bruxism is the characteristic squeaky or crunching sound that occurs as a result of rubbing of the teeth.

Limitations of our study included its retrospective nature, limited case of numbers and lack of electroencephalography to completely eliminate a focal seizure during AB.

To our knowledge, this report is the first description of AB associated with structural forebrain disease in dogs. Although limited by the low number of cases included, its retrospective nature, lack of a definitive diagnosis in 2 cases, and the neuroanatomical distribution of the lesions based on analysis of the historical and clinical information, we propose that the presence of AB associated with other neurological indications of forebrain disease should raise clinical suspicion for forebrain involvement in dogs and more specifically of a lesion affecting the diencephalon, frontal cortex or both. Additional studies of AB in dogs should be considered including its relationship with diencephalic disease and its clinical relevance.

## CONFLICT OF INTEREST DECLARATION

Authors declare no conflict of interest.

## OFF‐LABEL ANTIMICROBIAL DECLARATION

Authors declare no off‐label use of antimicrobials.

## INSTITUTIONAL ANIMAL CARE AND USE COMMITTEE (IACUC) OR OTHER APPROVAL DECLARATION

Authors declare no IACUC or other approval was needed.

## HUMAN ETHICS APPROVAL DECLARATION

Authors declare human ethics approval was not needed for this study.

## Supporting information


**Appendix S1**. Supporting Information

**Video S1**. Video of AB in a dog with MUO. Note the characteristic squeaky or crunching sound of rubbing of the teeth

**Video S2**. Video of AB in a dog with congenital brain anomaly and MUO
