# Supplementary material for: Bruxism in awake dogs as a clinical sign of forebrain disease: 4 cases
Source: J Vet Intern Med. 2022 Nov 2;36(6):2132–41. doi: 10.1111/jvim.16570 (PMC9708440; doi:10.1111/jvim.16570)
Supplement: Supplementary file 1 — Appendix S1. Supporting Information [file JVIM-36-2132-s003.pdf]

S1. Database of canine cases with episodic awake bruxism

Signalment, onset of signs and presenting complaints, clinical findings, diagnostic test results, clinical diagnosis, treatment, outcome, of the four canine cases that manifested episodic awake bruxism.

C	Signalment	Onset of signs and Presenting complaints	Clinical findings	Diagnostic test results	Clinical diagnosis	Treatment	Outcome
1	4yo (21kg)<br>FN<br>Labrador	**Onset**: <24h, progressive<br>**Presenting complaints**:<br>Urinary/faecal accidents<br>L-sided limb knuckling<br>L Head turn<br>L Body turn	**CE**: Tachypnoea<br><br>**NE**: L head turn; L head tilt L pleurothotonus; L hemiparesis; L circling; Absent postural reactions on L PL and TL; L absent menace response; L Horner's syndrome (miosis, third eyelid protrusion); L decreased PLR; Episodic head tremor; Episodic bruxism<br><br>**NAL**: L forebrain (suspected diencephalon)	**CBC, SB, BA**: WNL<br>**Infectious disease tests**:<br>IFA – *Toxoplasma gondii* IgG/IgM: negative<br>IFA – *Neospora caninum* IgG: negative<br>ELISA – *Anaplasma* spp., *Borrelia* spp., *Ehrlichia* spp., *Dirofilaria* spp.: negative<br>**Head MRI**:<br>(a) T2W and FLAIR hyperintense, T1W hypointense, poorly and heterogeneously contrast enhancing intra-axial lesion occupying a large area of the brainstem, extending from the level of the pons and occupying the majority of the midbrain (right-side more severely affected) to the level of the thalamus/hypothalamus.<br>(b) adjacent meningeal contrast enhancement<br>(c) Mild midline shift to L at the level of interthalamic adhesion;<br>(d) mild rostrodorsal displacement of the caudoventral aspect of interthalamic adhesion.<br>**CSF analysis** (cisternal):<br>mixed pleocytosis (TNCC: 3114/µL, RI: 0-5; total microproteins: 223 mg/dl (RI< 36 mg/dl).<br>**CSF culture:** negative	MUO	Dexamethasone<br>0.3 mg/kg IV q24h<br><br>Cytarabine<br>100mg/m^2 SC<br>q12h x 2 doses<br><br>Clindamycin<br>12.5 mg/kg IV<br>q12h\<br><br>Levetiracetam<br>30 mg/kg IV q8h<br><br>Paracetamol<br>10 mg/kg IV q8h<br><br>Maropitant<br>1 mg/kg IV q24h<br><br>IVFT	Euthanised due to non-improvement.
2	6yo (27kg)<br>FN Boxer	**Onset**: 2m, progressive<br>**Presenting complaints**:<br>Episodic teeth grinding<br>Episodic head tremor<br>Ignoring right-side<br>Lethargy	**CE**: Tachypnoea<br><br>**NE**: L head turn; L head tilt; L circling; decreased menace response (R>L); episodic	**CBC, SB, BA**: WNL<br>**Head MRI:**<br>(a) large clearly defined, rounded T2W hyperintense, T1W hypointense, FLAIR hyperintense with slightly hypointense centre, minimally contrast enhancing, intra-axial mass	Brain mass (susp. Left forebrain glioma)	Prednisolone<br>2 mg/kg PO q24h<br><br>Maropitant<br>1 mg/kg PO q24h	Improved initially but then deteriorated and was euthanised 2m post discharge.

		Reduced barking	bruxism; R hemi-neglect syndrome<br><br>**NAL**: L forebrain (suspected diencephalon)	lesion centred over the left internal capsule and piriform lobe, extending into the frontal lobe and causing marked mass effect on the left thalamus.<br>(b) moderate midline and third ventricle shift to the right.<br>(c) left lateral ventricle compression.		Lomustine $65mg/m^2$ PO x 1 dose	
3	9mo (24kg) FE Great Dane	**Onset**: <24h, progressive<br>**Presenting complaints**:<br>Lethargy<br>Tripping over the door<br>Hypodipsia	**CE**: WNL.<br><br>**NE**: obtundation; ventroflexion of the neck; R head turn; R pleurothotonus; mild hypermetria in TLs; tendency to pace; episodic bruxism; facial grimace with pinned back ears and aggression when touching the head (headache)<br><br>**NAL**: R forebrain (suspected diencpehalon)	**CBC, SB, BA**:<br>ALB 36.2 g/L (RI: 26-35); ALP 194 U/L (RI: 21-102); CHOL 15.5 mmol/L (RI: 3.8-7); TRIG 1.31 mmol/L (RI: 0.57-1.14); CK 306 U/L (RI: 50-200); UREA 11.8 mmol/L (RI: 1.7-7.4); P 2.6 mmol/L (RI: 0.9-2); Na 190 mmol/L (RI: 139-154); Cl 148 mmol/L (RI: 102-118).<br>**Basal cortisol**: WNL.<br>**Urinalysis**: WNL.<br>**Head MRI**:<br>(a) absence of rostrum, genu and body of corpus callosum with only a small portion of the splenium visible.<br>(b) the lateral ventricles have upturned pointed corners (upturned bat sign).<br>(c) at the level of the thalami, the lateral ventricles are fused and there is absence of septum pellucidum.<br>(d) slightly rostral to the lamina terminalis, there is absence of the septum separating the ventral aspect of the L and R frontal lobes.<br>(e) The pituitary gland is subjectively small, with mildly increased CSF-accumulation in the sella turcica. On post contrast imaging a T1W hypointense V-shape of the hypothalamus is visible.<br>(f) The dorsal aspect of the cerebellar vermis has an s-shaped conformation with mild caudal herniation of the dorsal aspect of the vermis at the foramen magnum likely secondary to the occipital bone dysplasia<br>**Abdominal u/s:** WNL.<br>**Thoracic radiographs**: WNL	Congenital brain anomaly complex (corpus callosum hypoplasia; partial lobar holoprosencephaly; partial empty sella with V-shaped neurohypophysis; occipital dysplasia with mild partial cerebellar herniation)	Desmopressin acetate 16 µg/kg on the conjunctiva q12h<br><br>Potassium salts 2mEq per 4.5 kg body weight PO q24 (if needed)	Euthanised 15d post diagnosis due to deterioration (anorexia, lethargy, constant bruxism, head pressing, yawning, seizures/facial twitches, stupor episodes, ataxia, deterioration of hypernatremia). Post-mortem examination revealed concurrent MUO.

4	9.5yo<br>(24kg) MN<br>Mongrel	**Onset**: 4m, progressive<br>**Presenting complaints**:<br>Anxiety, aggression<br>Reverse sneezing<br>Sneezing (non-productive)<br>Episodic teeth grinding<br>Tachypnoea episodes<br>Absence of barking, started howling<br>Urinary/faecal accidents	**CE**: Tachypnoea<br><br>**NE**: episodic bruxism; anxiety/pain; resistant on lateral manipulation of neck<br><br>**NAL**: forebrain	**CBC, SB, BA:** CK 253 U/L (RI: 50-200).<br>**Ammonia**: WNL.<br>**Head CT**:<br>(a) a well-circumscribed, irregularly marginated and strongly but heterogeneously contrast-enhancing intra-ventricular mass lesion with focal areas of hypoattenuation located in the region of the third ventricle, closely associated with the medial aspect of the right lateral ventricle.<br>(b) mild enlargement of the lateral ventricles and dilation of the olfactory recesses. Asymmetry of the lateral ventricles, with mild mass effect/distortion of the right lateral ventricle. There is dilation of the mesencephalic aqueduct.<br>(c) The mass is causing compression of the dorsal aspect of the right thalamus and midbrain and mild midline shift towards the left.<br>(d) moderate caudal transtentorial cerebral herniation causing compression of the rostral cerebellum.<br>(e) Mild foraminal cerebellar herniation.	Intraventricular brain mass (susp. choroid plexus tumour)	Prednisolone<br>1 mg/kg PO q12h<br><br>Sucralfate<br>1.5 g/dog PO q8h<br><br>Omeprazole<br>1 mg/kg PO q12h	Euthanised.

ALB: albumin
ALP: alkaline phosphatase
BA: pre-prandial bile acids
C: case number
CBC: complete blood count
CE: clinical examination
CHOL: cholesterol
CK: creatine kinase
CSF: cerebrospinal fluid
CT: computed tomography
FE: female entire
FN: female neutered
h: hours
IFA: indirect immunofluorescence
IV: intravenous
L: left
m: months
ME: male entire
MN: male neutered
MRI: magnetic resonance imaging
MUO: Meningoencephalitis of Unknown Origin
NAL: Neuroanatomical localisation
NE: neurological examination
PL: pelvic limb
PLR: pupillary light reflex
PO: per os
R: right
RI: reference intervals
SB: serum biochemistry
SC: subcutaneous
TL: thoracic limb
TNCC: total nucleated cell count
TRIG: triglycerides
WNL: within normal limits
Y: yes
